# All-cause Dementia Prediction by Machine Learning: The Health, Aging, and Body Composition Study

**DOI:** 10.1093/geroni/igaa057.1575

**Published:** 2020-12-16

**Authors:** Chenkai Wu, Xurui Jin

**Affiliations:** 1 Duke Kunshan University, Kunshan, Jiangsu, China; 2 Duke Kunshan University, Kunshan, China

## Abstract

There are several shortcomings of the currently available risk prediction models for dementia. We developed a risk prediction model for dementia using machine-learning approach and compared its performance with traditional approaches. Data were from the Health, Aging, and Body Composition Study, comprising 3,075 older adults (at least 70 years). Dementia was defined as (1) use of a prescribed dementia medication, (2) adjudicated dementia diagnosis, or (3) a race-stratified cognitive decline>1.5 SDs from the baseline mean. We selected 275 predictors collected from questionnaires, imaging data, performance testing, and biospecimen. We used random survival forest (RSF) to build the full model and rank the importance of predictors. Subsequently, we built parsimonious models with top-20 predictors using RSF and Cox regression. A dementia risk score was developed using top-ranked variables. We used the C-statistic for performance evaluation. Over a median of 11.4 years of follow-up, 659 dementias (21.4%) occurred. The RSF model (both including all and top-20 variables) showed a higher C-statistic than the regression model. Digit symbol score, physical performance battery, finger tapping score, weight change since age 50, serum adiponectin, and APOE genotype were the top-6 variables. We created a dementia risk score (0-10) using the top-6 variables. A 1-unit increase in the risk score was associated with an 8% higher risk of dementia. The risk score demonstrated good discrimination (C-statistic=0.75). Machine learning methods offered improvement over traditional approaches in predicting dementia. The risk prediction score derived from a parsimonious model had good prediction performance.

